# Melanonychia – Clues for a Correct Diagnosis

**DOI:** 10.7759/cureus.6621

**Published:** 2020-01-10

**Authors:** Teodora C Gradinaru, Mara Mihai, Cristina Beiu, Tiberiu Tebeica, Calin Giurcaneanu

**Affiliations:** 1 Dermatology, Elias Emergency University Hospital, Bucharest, ROU; 2 Oncologic Dermatology, Elias Emergency University Hospital, Carol Davila University of Medicine and Pharmacy, Bucharest, ROU; 3 Dermatopathology, Dr. Leventer Centre, Bucharest, ROU

**Keywords:** melanonychia, dermoscopy, nail pigmentation, melanoma, nail diseases

## Abstract

Melanonychia represents a brown to black discoloration of the nail plate that may be induced by benign or malignant causes. Two main mechanisms are involved in the appearance of melanonychias, i.e., melanocytic activation and melanocytic hyperplasia. The distinction between the two can be made based on the medical history of the patient, the clinical picture, dermoscopy, and histopathological examination and is essential for the adequate management of the patient. We review the main causes of melanonychia, with emphasis on the clues to the diagnosis of subungual melanoma.

## Introduction and background

Melanonychias represent brown to black discolorations of the nail plate. Longitudinal melanonychia is the most common form of presentation, whereas transversal and total melanonychia are rarely encountered. Two main mechanisms are involved in the development of melanonychia: hypermelanosis or melanocytic activation and melanocytic hyperplasia [1]. The aim of this paper is to review and summarize the clinical, dermoscopic, and histopathological findings for the main causes of melanonychia, highlighting that early diagnosis is of crucial importance for the management and prognosis of subungual melanoma. All clinical and dermoscopic images included in the review section of the article were taken in the Department of Oncologic Dermatology of Emergency University Hospital “Elias” in Bucharest. Clinical photographs were taken using a digital camera (Nikon D3300; Nikon Corporation, Tokyo, Japan). Dermoscopic images were acquired using a digital videodermoscopy system (FotoFinder, Bad Birnbach, Germany). Histopathological images were provided by “Dr. Leventer Centre” in Bucharest, where histological samples were prepared and interpreted. All patients have given written informed consent. 

## Review

The terms hypermelanosis or melanocytic activation refer to an increased melanin production, which leads to the pigmentation of the nail matrix epithelium and nail plate. On histological examination, the number of melanocytes in these sites is within normal limits [1]. The epithelial hyperpigmentation is not evident on hematoxylin-eosin staining but can be observed on Fontana-Masson stained sections. Immunohistochemistry studies using Melan-A, HMB-45, S100, and Ki67 antibodies are of great help in establishing the diagnosis.

**Figure 1:**
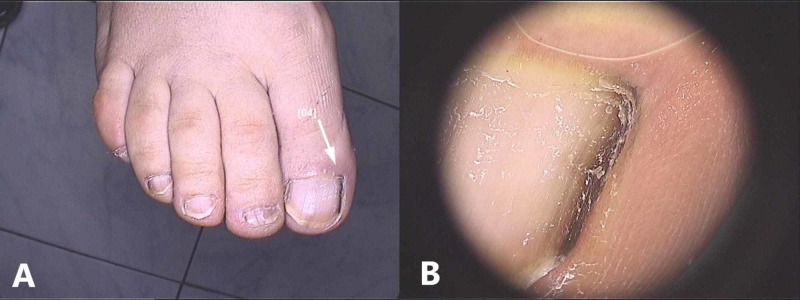
Hypermelanosis manifesting as longitudinal brown melanonychia: (A) Clinical picture (B) Dermoscopic appearance

Clinically, hypermelanosis manifests as an asymptomatic longitudinal gray, brown, or black band of the nail plate that starts from the nail matrix and ends at the tip of the nail plate (Figure 1). Melanocytic activation usually involves multiple nails [2].

A wide variety of factors may induce hypermelanosis. Physiological causes include pregnancy and racial melanonychia. In the latter, the width, as well as the number of pigmented nail bands may increase with age. Among the iatrogenic causes of melanonychia, the most common are phototherapy, X-ray exposure, and medication (antimalarial therapy, hydroxyurea, busulphan, bleomycin, doxorubicin, cyclophosphamide, 5-fluorouracil). These are most frequently associated with transverse melanonychia [2]. In the majority of iatrogenic melanonychia cases, the pigmentation develops 3-8 weeks after the initiation of treatment and fades away 6-8 weeks following treatment cessation [3]. Melanonychia is also a frequent finding in a series of dermatoses like psoriasis, lichen planus, and Hallopeau acrodermatitis. Patients generally present one light brown longitudinal band, which appears after the resolution of the inflammatory process [2]. 

Systemic causes of melanonychia include endocrine disorders (Addison disease, Cushing disease, acromegaly, hyperthyroidism), hemosiderosis, hyperbilirubinemia, porphyria, and genetic syndromes (Laugier-Hunziker, Touraine, and Peutz-Jeghers syndromes) [2]. Syndrome-associated melanonychia manifests as multiple longitudinal pigmented bands of the nail plate and multiple pigmented macules on the lips and in the oral cavity. 

**Figure 2:**
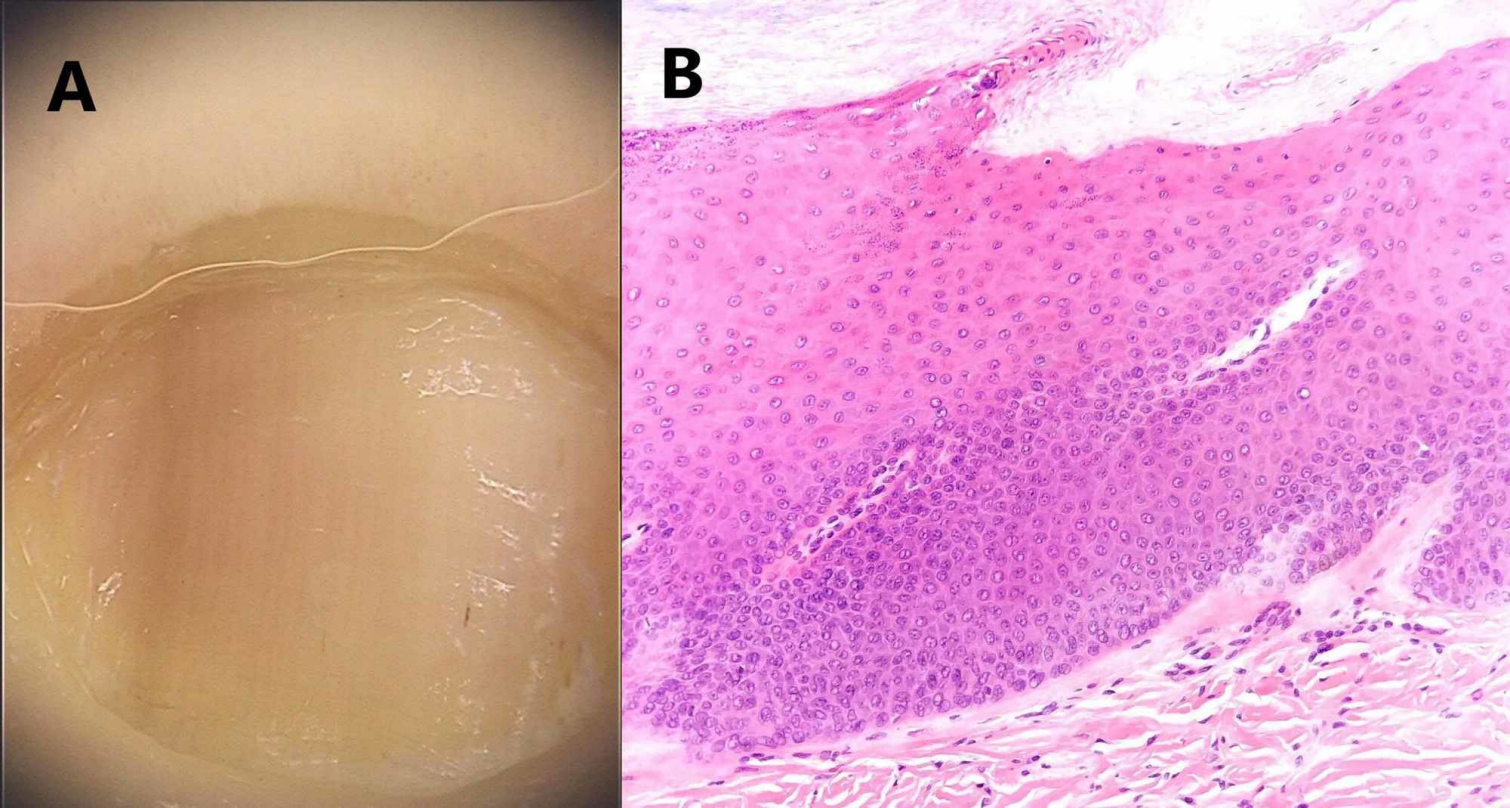
Frictional longitudinal melanonychia of the fifth toe: (A) Macroscopic image. (B) Histological image: while no melanocytes are detected on hematoxylin-eosin stained longitudinal nail biopsy, Melan A staining reveals dispersed melanocytes with dendritic cytology

Melanonychias induced by local causes, such as onychophagia and onychotillomania, are usually accompanied by Beau’s lines, nail thinning, longitudinal striations, onychorrhexis, splitting of the distal nail margin, cuticular damage, or crusts. Frictional trauma typically generates pigmentation of the medial part of the hallux and lateral parts of the fifth and fourth toes (Figure 2). Infections, both bacterial (especially *Proteus mirabilis*) and dermatophytic (*Trichophyton rubrum nigricans*) often lead to melanocytic activation due to the inflammatory response [2]. In this case, the brown longitudinal band is accompanied by a subungual hyperkeratosis, yellow or brown crusts, nail dystrophy, and occasionally a reddish hue due to traumatic hemorrhages but no melanin granules [4].

Dermoscopically, nail hypermelanosis appears as a brown or gray homogenous band. The lines can be regular or not. Red dots may also be observed, suggesting blood extravasation or splinter hemorrhages due to trauma [2].

The histopathologic diagnosis of ungual hypermelanosis is based on the normal number and appearance of melanocytes, which are located in the suprabasal layer and the absence of mitoses. In case of onychomicosis, the pseudohyphae and spores are easily detected on periodic acid-Schiff staining. Bacteria can also be revealed using histochemical stains [5]. 

**Figure 3:**
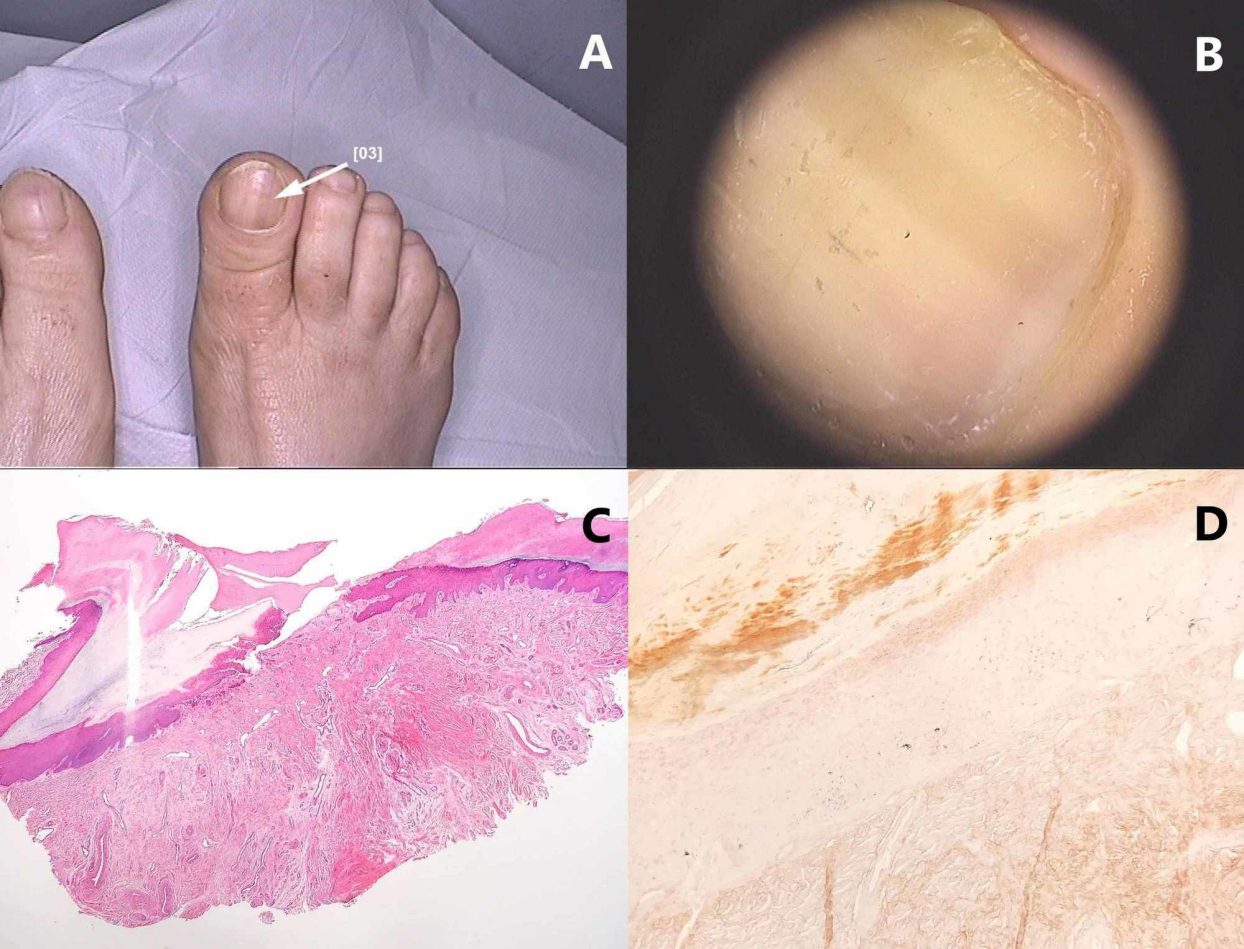
Nail apparatus lentigo: (A) Macroscopic image. (B) Dermoscopic image that reveals a longitudinal, light brown band. (C) Longitudinal nail biopsy of a nail matrix lentigo – melanocytes are not observed on hematoxylin-eosin staining. (D) Fontana-Masson stained sections show minimal pigmentation

Melanocytic hyperplasia designates the proliferation of the melanocytes of the nail matrix and nail plate [5]. The process can be either benign (lentigo, nevus), or malignant (melanoma). Subungual lentigo manifests as a brown longitudinal band (Figure 3). Over time, additional bands may develop. While the melanocytes of nevi characteristically form at least one nest, lentigines are composed of melanocytes that do not form nests [6]. In lentigines, the melanocytes are located mainly in the basal layer and can reach 10-31 melanocytes/mm, in contrast with the normal matrix melanocytes, which are present above the basal layer [2, 7]. They present fine melanin granules and no cytological atypia. Melanophages may be observed in the dermis. The whole matrix epithelium, as well as the nail plate are pigmented [8].

**Figure 4:**
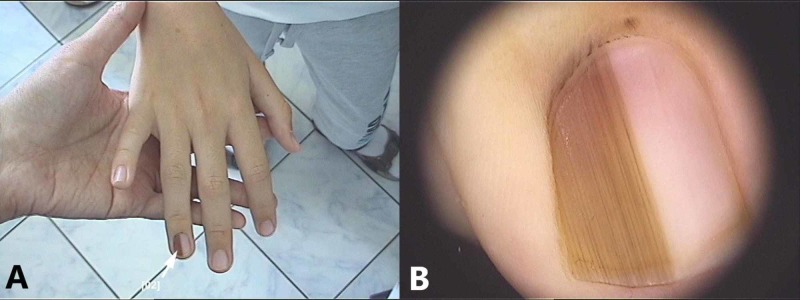
Congenital ungual nevus: (A) Clinical picture showing light and dark longitudinal brown bands and periungual pigmentation; (B) Dermoscopic appearance

Dermoscopically, ungual nevi display a brown background, brown to black parallel lines with the same width and regular spaces. Congenital nevi usually have a triangular aspect, proximally wider, and the Pseudo Hutchinson’s sign is frequently found [4]. Pseudo Hutchinson`s sign indicates the pigmentation seen due to the transparency of the cuticle and is also found in other benign situations, such as Peutz-Jeghers syndrome, Laugier-Hunziker syndrome, trauma, and medication use (zidovudine, minocycline) [2]. Moreover, they often exhibit periungual pigmentation that can involve the hyponichium and proximal nail fold (Figure 4) [2]. Dermoscopy of regressive ungual nevi reveals black spots over the pigmented bands, the latter having regular shapes and sizes (<0.1 mm). In the long term, due to a decrease in the activity of melanocytes, the black spots disappear and the intensity of the melanonychia diminishes [4].

As mentioned previously, the histopathologic examination of nail nevi shows the proliferation of melanocytes that organize into nests. Nevi occurring in the nail unit usually display a lentiginous pattern and a tendency of pagetoid spread. Likewise, the majority of matrix melanocytic nevi are junctional nevi. Compound matrix nevi are infrequent [9]. Nuclear atypia should be interpreted as a sign of malignancy even if it is minimal and only noticed in a few cells [10]. Pagetoid spread, hyperchromasia of melanocytes, and interface inflammation should also be regarded with great caution, the differentiation between atypical nevi and early melanoma representing a real challenge even for experienced pathologists [10-11]. Immunohistochemical stains help in the differential diagnosis between benign and malignant melanocytic proliferation, although S-100 staining is sometimes lost in ungual melanoma [9].

**Figure 5:**
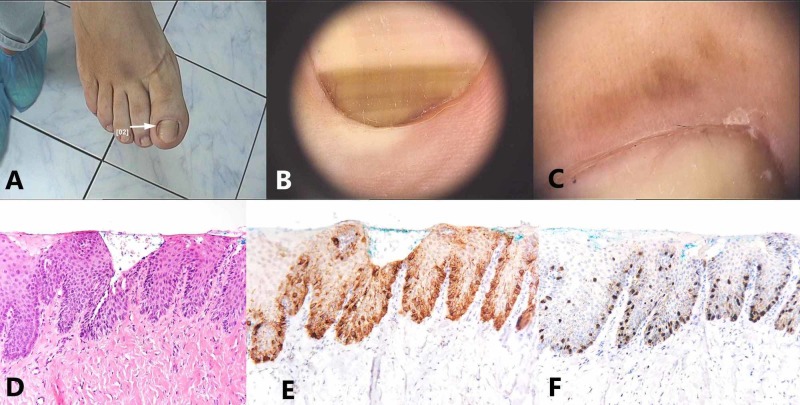
Subungual in situ melanoma: (A) Macroscopic image. (B, C) Dermoscopic images showing an aspect of longitudinal melanonychia composed of bands of different width, spacing and color, accompanied by Hutchinson’s sign; (D) Longitudinal biopsy – hematoxylin–eosin stain. (E,F) Melan A stain showing a lentiginous proliferation of atypical melanocytes

**Figure 6:**
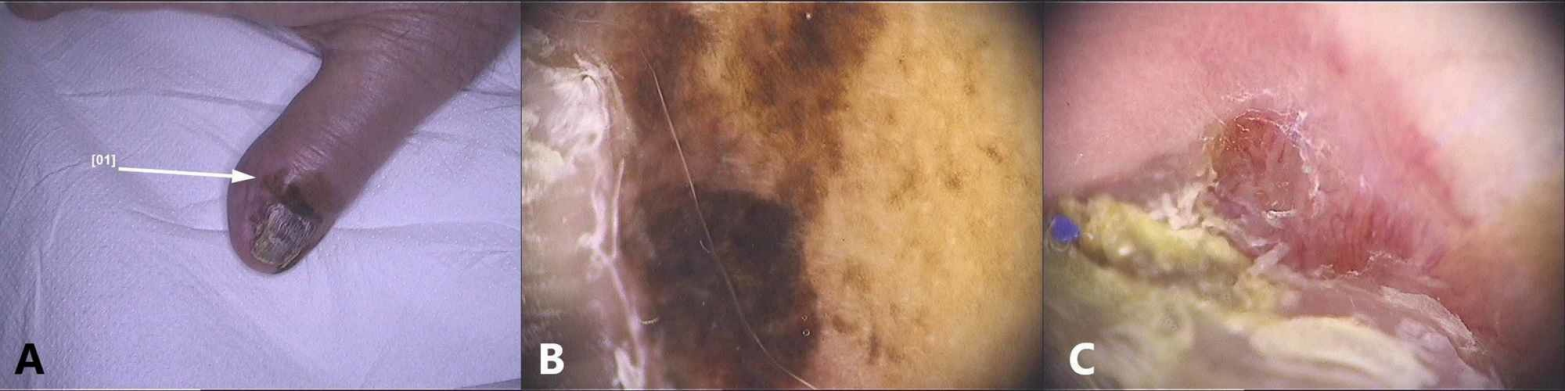
Subungual melanoma: (A) Macroscopic image, (B) Dermoscopic findings - pigmentation of the eponichium, (C) Nail distrophy and vessels

Longitudinal melanonychia is the main sign of subungual melanoma, considering that 2/3-3/4 of subungual melanoma clinically present as longitudinal melanonychia [12]. Melanonychia in melanoma has several characteristic features. The width of melanonychia is a very important marker of melanoma. A variation in width is generally observed. It may start as a thin longitudinal melanonychia and gradually evolve to total melanonychia. Its shape is also important. The longitudinal pigmented band may take a pyramidal or a triangular shape. The widest part is near the cuticle, which implies a progressive growth [2]. Subungual melanoma can vary in color from light brown to black. As a rule, only one finger is involved (Figure 5). Other signs of melanoma include nail dystrophy, the presence of Hutchinson’s sign, pain, bleeding, and secondary infection (Figure 6). Hutchinson`s sign is defined by the extension of the brown-black pigment from the matrix, nail bed, and nail plate onto the cuticle and lateral and proximal nail folds. Micro Hutchinson sign refers to the pigmentation of the cuticle that is not macroscopically visible and can only be observed by dermatoscopic examination [2].

Dermoscopic examination of subungual melanomas shows heterogeneous longitudinal black or brown lines of irregular thickness and spacing, with lost parallelism. Pigment granules can be observed. Hutchinson`s and micro Hutchinson’s signs are often present. Clues to the origin of the pigment should be sought. If the pigment is present on the dorsal part of the free edge of the nail plate, it originates in the proximal matrix. On the contrary, if the pigment is located on the ventral part of the free edge of the nail plate, it derives from the distal matrix [4].

The ABCDEF rule for the diagnosis of subungual melanoma is profoundly different from that of cutaneous melanoma and comprises the following criteria: A - age and race (40-70 years, African Americans, Asians), B - brown to black longitudinal band, width over 3 mm, irregular blurred borders, C - change, D - digit (thumb > big toe > index finger), E - extension of pigmentation, Hutchinson’s sign, F - family or personal history of melanoma or dysplastic nevi [6, 13-14].

The histologic subtypes of subungual melanoma are acral lentiginous melanoma, superficial spreading melanoma, and nodular melanoma, the former being the most frequent [13, 15]. Melanoma generally originates in the matrix and rarely in the nail bed. The histological features that suggest malignancy are the asymmetry of the lesions, the infiltrative margins, the markedly increased number of melanocytes in the basal layer (upto 39-136/mm), and the suprabasal layers with a high propensity to form compact aggregates, the presence of cytological atypia, and dermal inflammation [16]. Malignant melanocytes have large, atypical nuclei, with increased mitotic activity or maybe multinucleated. Identification of melanocytes in the nail plate is diagnostic for melanoma. Invasive subungual melanoma also exhibits irregularly dispersed dermal nests composed of atypical melanocytes. Similar to skin melanoma, the dermal component of subungual melanoma shows loss of maturation. As the papillary dermis and subcutis are absent in the nail apparatus, the correlation between thickness of the lesion and outcome differs from that of sin melanoma [17].

**Figure 7:**
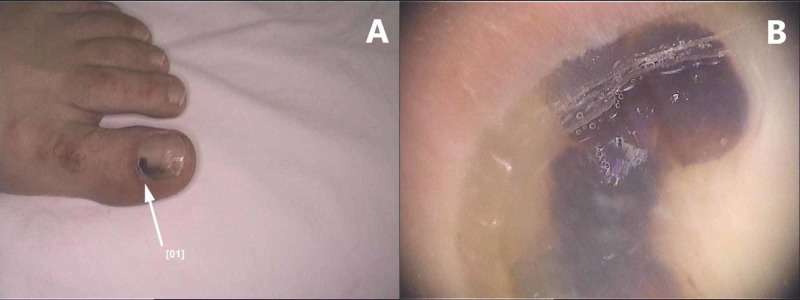
Subungual hematoma: (A) Macroscopic appearance, (B) Dermoscopy image showing a dark red homogeneous pigmentation that does not extend to the free edge

The most important differential diagnosis for subungual melanoma is nail hematoma, one of the commonest causes of brown-black pigmentation of the nail plate. On dermoscopic examination, a red-violet-brown globular pattern is seen along the nail plate, as well as a homogeneous pigmentation of the nail, which vanishes in the periphery. There are no longitudinal lines as those that characterize true melanonychia (Figure 7). In addition, the lesion does not reach the free edge of the nail plate. Hemostix test is positive [4, 13]. As the nail grows, the area of discoloration moves towards the free edge of the nail and is eventually cut out.

Bowen’s disease may occasionally affect the nail unit and initially present as longitudinal melanonychia. Human papillomavirus infection, trauma, exposure to radiation or arsenic are predisposing factors [18]. Histopathologically, it is characterized by thickened nail bed epithelium with atypical keratinocytes with bizarre mitotic nuclei and dyskeratosis.

## Conclusions

In conclusion, melanonychia often poses diagnostic problems. Clinicians should be aware of its potential causes and maintain a high index of suspicion, which is essential for early diagnosis of subungual melanoma. The steps towards establishing the correct diagnosis are a meticulous anamnesis, a thorough physical and dermoscopic examination, and performing a biopsy from any suspicious lesion, followed by a comprehensive clinical-pathological correlation.
